# Fatal SARS in X-Linked Lymphoproliferative Disease Type 1: A Case Report

**DOI:** 10.3389/fped.2022.794110

**Published:** 2022-04-14

**Authors:** Ming Hin Chung, Gilbert T. Chua, Daniel Leung, Koon Wing Chan, John Nicholls, Yu Lung Lau

**Affiliations:** ^1^Department of Pediatrics and Adolescent Medicine, LKS Faculty of Medicine, The University of Hong Kong, Hong Kong, Hong Kong SAR, China; ^2^Department of Pediatrics and Adolescent Medicine, Li Ka Shing Faculty of Medicine, The University of Hong Kong, Hong Kong, Hong Kong SAR, China; ^3^Department of Pathology, Li Ka Shing Faculty of Medicine, The University of Hong Kong, Hong Kong, Hong Kong SAR, China

**Keywords:** COVID-19, X-linked lymphoproliferative disease type 1 (XLP1), severe acute respiratory syndrome (SARS), agammaglobulinemia, inborn error of immunity

## Abstract

X-linked lymphoproliferative disease (XLP1) is an inborn error of immunity (IEI) with severe immune dysregulation caused by a mutation in the *SH2D1A* gene resulting in the absence or dysfunction of signaling lymphocytic activation molecule (SLAM)-associated protein (SAP). The severe acute respiratory syndrome (SARS) caused by SARS-coronavirus (CoV), a highly pathogenic CoV, has been shown to only cause mild diseases in Asian children. We report on a 5-year-old Nepalese boy with agammaglobulinemia and probable SARS who died of diffuse alveolar damage 22 days after admission amid the SARS outbreak. The index patient and his younger brother were genetically confirmed to have XLP1. In the current coronavirus disease 2019 (COVID-19) pandemic, most children also had mild disease only. Children with severe COVID-19 would warrant investigations for underlying IEI, particularly along the pathways leading to immune dysregulation.

## Introduction

X-linked lymphoproliferative disease (XLP1) is an inborn error of immunity (IEI) characterized by primary hemophagocytic lymphohistiocytosis, fatal infectious mononucleosis, and B-cell malignancies associated with Epstein-Barr virus infection and dysgammaglobulinemia. XLP1 is caused by a mutation in the *SH2D1A* gene resulting in the absence or dysfunction of signaling lymphocytic activation molecules (SLAM)-associated protein (SAP), which is commonly expressed in natural killer (NK) cells, killer T (NKT) cells, and CD4 and CD8+ T lymphocytes. Defective expression of SAP in patients with XLP1 leads to increased susceptibility to viral infections. However, to our knowledge, there is no research on how underlying XLP1 in patients would affect the prognosis of the severe acute respiratory syndrome (SARS) or coronavirus disease 2019 (COVID-19) infection. Here, we present an extremely rare case of fatal SARS in a patient with uncontrolled and excessive inflammation and underlying XLP1.

## Case Description

**Figure 1:**
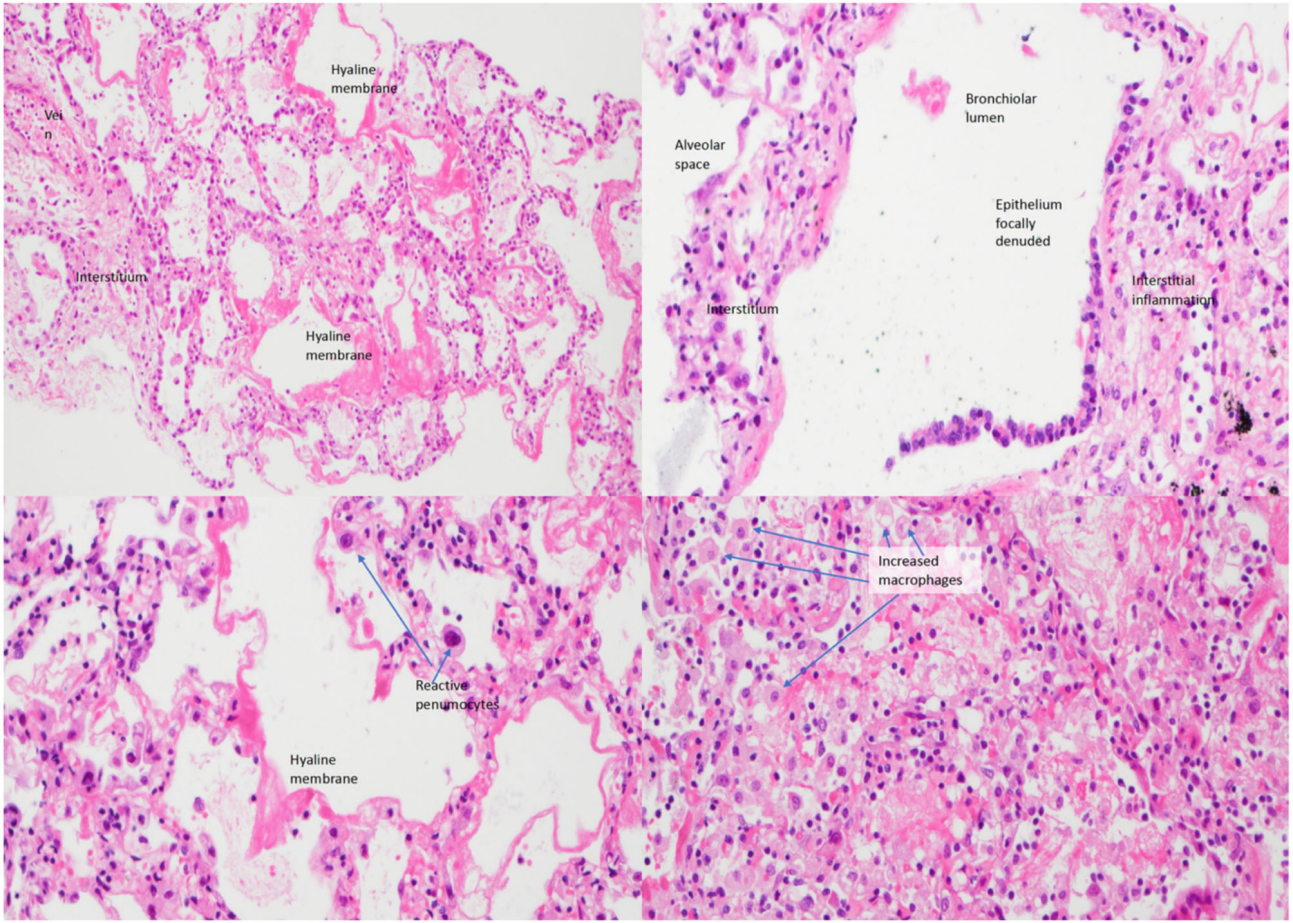
Lung biopsy of the patient under low power (left upper figure), medium power (right upper), and high power (left lower and right lower) showing interstitial inflammation, infiltration of macrophages and reactive pneumocytes with evidence of diffuse alveolar damage.

A 5-year-old Nepalese boy with recurrent pneumococcal and adenovirus type 6 pneumonia was found to have agammaglobulinemia in early 2003. He was born of non-consanguineous parents with no significant family history. He had a history of recurrent otitis media with perforations and persistent cough with yellow sputum. His baseline immunoglobulin subsets showed low serum levels of IgG (<33 mg/d, reference range 617–1,445 mg/dl), IgA (<6.7 mg/dl, reference range 58–240 mg/dl), and IgM (16.0 mg/dl, reference range 64–249 mg/dl). Regular replacement of intravenous immunoglobulin (IVIG, 0.5 g/kg/dose) was given. His baseline lymphocyte subset showed increased T-cells (8,701/ul, reference range 1,500–2,900/ul), decreased natural killer (NK) cells (254/ul, reference range 300-600/ul), reversed CD4 to CD8 ratio (0.57, reference range 1.1–2), and normal B-cell level (1117/ul, reference range 500–1,200/ul). He was admitted in March 2003, at the peak of the severe acute respiratory syndrome (SARS) epidemic in Hong Kong (1), with fever, cough, and coryzal symptoms but without respiratory distress. The nasopharyngeal aspirate (NPA) polymerase chain reaction (PCR) on admission tested positive for SARS-CoV. Chest X-ray revealed patchy bilateral infiltrates and pleural effusion. He did not respond to multiple antibiotics, intravenous ribavirin, high-dose intravenous hydrocortisone 8 mg/kg/day, and IVIG 0.84 g/kg/dose. Twelve days after admission, he was transferred to the pediatric intensive care unit because of progressive respiratory failure and bilateral pleural effusion. Bronchoalveolar lavage and pleural fluid for extensive microbiological workup were unrevealing. He developed massive pleural effusion, required high inotropic support, and progressed to multi-organ failure. He passed away 22 days after admission. Perimortem lung biopsy showed focally denuded alveolar epithelium and diffuse alveolar damage (Figure 1), compatible with the pathology of fatal SARS infection (2). There were interstitial inflammation, infiltration of macrophages and reactive pneumocytes, but no neutrophil, B-cell, and plasma cell accumulation, and no viral antigens were identified in the lung biopsy, suggesting an immune-mediated pathology rather than a direct invasion of SARS-CoV.

**Figure 2:**
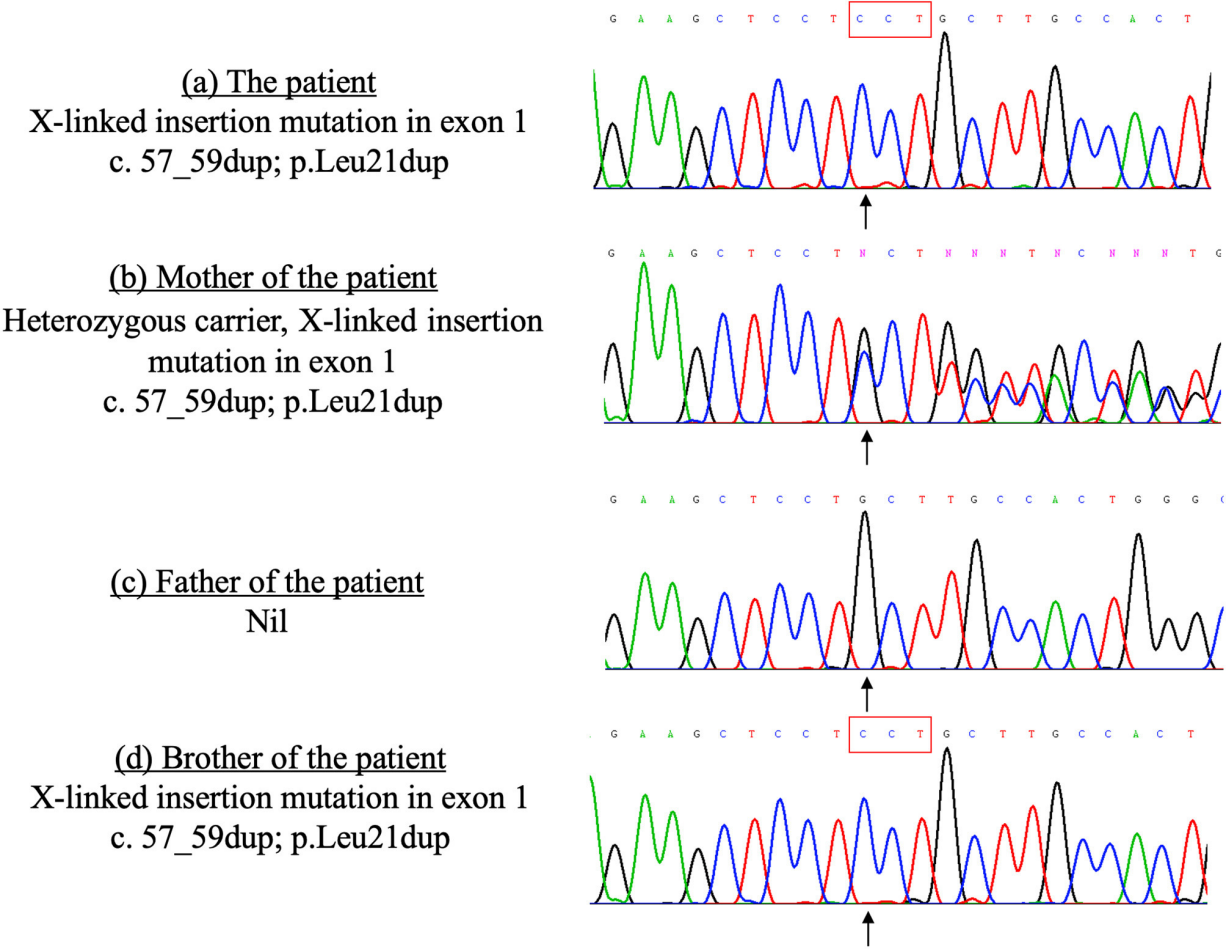
Sanger sequencing of the *SH2D1A* gene of the patient and his family members done in 2010. The patient and his brother carry the same X-linked mutation in the exon 1 of the *SH2D1A* gene, which was inherited from their mother. **(a)** The patient X-linked insertion mutation in exon 1 c.57_59dup; p.Leu21dup. **(b)** Mother of the patient Heterozygous carrier, X-linked insertion mutation in exon 1 c.57_59dup; p.Leu21dup. **(c)** Father of the patient Nil. **(d)** Brother of the patient X-linked insertion mutation in exon 1 c.57_59dup; p.Leu21dup.

The patient's younger brother was born 3 years after the patient's death with no significant health concerns. His lymphocyte subset and immunoglobulin profile were similar to that of his deceased elder brother, which showed increased T-cells (4,112/ul, reference range 1,500–2,900/ul), decreased NK cells (212 /ul, reference range 300–600/ul), and normal B cells (975/ul, reference range 500–1,200/ul). Serum IgG level gradually dropped from 603 to 350 mg/dl (reference range 251–724 mg/dl) 4 months after birth. A genetic test of siblings by whole-exome sequencing revealed a pathological hemizygous mutation in the *SH2D1A* gene (X-linked insertion mutation in exon 1, c.57_59dup; p.Leu21dup, reference sequence LRG_106), confirming the diagnosis of X-linked lymphoproliferative disease type 1 (XLP1), with the mother being a carrier (Figure 2). In retrospect, the absence of B-cells and plasma cell infiltration in the index patient's lung biopsy is also compatible with findings of other documented cases of XLP (3). The younger brother was treated with regular IVIG replacement and later received hematopoietic stem cell transplantation.

## Discussion

Although there has been no direct evidence that subjects with severe SARS infection could be indicative of an inborn error of immunity (IEI), genetic polymorphism involving the innate immunity, such as the expression of low-MBL-producing genotypes, was associated with an increased risk of severe SARS (4). To our understanding, there have been no reported cases of SARS infection in patients with XLP1 leading to death. Although the diagnosis of SARS was not in doubt in this patient with XLP1 because of the positive PCR, the lung biopsy did not show positive antigen, which is not unexpected, as viral antigen disappears after 2 weeks.

The underlying pathophysiology of such a severe phenotype could be explained by the immune dysregulation of XLP1 leading to an uncontrolled inflammatory response triggered by SARS-CoV infection. XLP1 is characterized by defective expression of SAP leading to increased susceptibility to viral infection and uncontrolled inflammation. First, the lack of SAP expression in CD4+ T cells leads to the defective maturation of terminal B-cells and the development of germinal centers for antibody production, causing hypogammaglobulinemia. Second, the deficiency of SAP in patients with XLP1 also leads to the defective production of proapoptotic molecules, resulting in an intrinsic defect in T-cell receptor restimulation-induced cell death (TCR RICD). This causes the defective apoptosis of T cells and excessive and lethal accumulation of activated CD8+ T cells, in particular, in Epstein-Barr virus infections (5). Third, studies have shown that patient with SARS with severe phenotypes, compared to those with milder conditions, had significantly higher levels of pro-inflammatory cytokines, such as interleukin 1 (IL-1), IL-6, and IFN-gamma, and inflammatory chemokines such as CCL3, CCL5, CCL2, and CXCL10, and significantly lower anti-inflammatory cytokines, in particular, IL-10 (6). In XLP1, SAP deficiency causes inadequate IL-10 production, which may result in excessive maturation of macrophages, leading to uncontrolled and excessive inflammation (7).

Seventeen years after the SARS outbreak, the COVID-19 pandemic resulted in over six million deaths in 2 years (8). SARS-CoV2 shares almost 80% of the genome with SARS-CoV, and patients with SARS or COVID-19 both developed lymphopenia with high levels of proinflammatory cytokines including interleukin (IL)-1b and IL-6 (9). Although COVID-19 has higher transmissibility than SARS (9), most children infected with SARS or COVID-19 have mild diseases (10, 11). The induction of a balance and effective cellular immunity is essential for the control of SARS-CoV2. For example, inborn errors of TLR3- and IRF7-dependent type 1 IFN immunity has been shown to be associated with life-threatening COVID-19 infection (12). It is possible that patients with XLP1 who have dysregulated T-cell activation may be at risk of severe COVID-19 infection because of adverse unregulated inflammatory responses (13). Study also suggested that younger male patients with IEI are more likely to suffer from severe COVID-19 infection and require intensive care admission because of differential levels of inflammatory mediators, T-cell responses, and virus-specific antibodies between infected males and females (14). However, certain patients with IEI tend to develop milder clinical presentation after COVID-19 infection. For example, COVID-19 infection in X-linked agammaglobulinemia patients are reported to have a milder phenotype than common variable immune deficiencies patients in small case series (15, 16). Therefore, patients with underlying immune dysregulation syndromes such as XLP1 could be at risk of severe diseases after SARS, and potentially COVID-19. Depending on the availability of resources in different jurisdictions, pediatricians should consider referral to immunologists and perform immunological investigations based on risk factors and severity of COVID-19 infection, such as complete blood count, immunoglobulin levels, lymphocyte subsets, cytokine profiles, and genetic analysis (17, 18). For severe pediatric patients with COVID-19, screening for underlying IEI is also recommended when available.

## Data Availability Statement

The original contributions presented in the study are included in the article/supplementary materials, further inquiries can be directed to the corresponding author.

## Author Contributions

MC wrote the first draft of the manuscript. GC and YL edited the manuscript. All authors contributed to manuscript revision, read, and approved the submitted version.

## Funding

YL received financial support from the Society for the Relief of Disabled Children to help children with IEI. However, society has no involvement in the collection, analysis, and interpretation of data, in the writing of the report, and in the decision to submit the article for publication.

## Conflict of Interest

The authors declare that the research was conducted in the absence of any commercial or financial relationships that could be construed as a potential conflict of interest.

## Publisher's Note

All claims expressed in this article are solely those of the authors and do not necessarily represent those of their affiliated organizations, or those of the publisher, the editors and the reviewers. Any product that may be evaluated in this article, or claim that may be made by its manufacturer, is not guaranteed or endorsed by the publisher.
